# Cyclosporin A increases recovery after spinal cord injury but does not improve myelination by oligodendrocyte progenitor cell transplantation

**DOI:** 10.1186/1471-2202-11-127

**Published:** 2010-10-12

**Authors:** He-Zuo Lü, Yan-Xia Wang, Jian-Sheng Zhou, Feng-Chao Wang, Jian-Guo Hu

**Affiliations:** 1 Central Laboratory, the First Affiliated Hospital of Bengbu Medical College, Anhui 233004, China; 2 Anhui Key Laboratory of Tissue Transplantation, Bengbu Medical College, Anhui 233004, China; 3 Department of Neurobiology, Shanghai Jiaotong University School of Medicine, Shanghai 200025, China; 4 Department of Clinical Laboratory Science, the First Affiliated Hospital of Bengbu Medical College, Anhui 233004, China

## Abstract

**Background:**

Transplantation of oligodendrocyte precursor cells (OPCs) is an attractive therapy for demyelinating diseases. Cyclosporin A (CsA) is one of the foremost immunosuppressive agents and has widespread use in tissue and cell transplantation. However, whether CsA affects survival and differentiation of engrafted OPCs *in vivo *is unknown. In this study, the effect of CsA on morphological, functional and immunological aspects, as well as survival and differentiation of engrafted OPCs in injured spinal cord was explored.

**Results:**

We transplanted green fluorescent protein (GFP) expressed OPCs (GFP-OPCs) into injured spinal cords of rats treated with or without CsA (10 mg/kg). Two weeks after cell transplantation, more GFP-positive cells were found in CsA-treated rats than that in vehicle-treated ones. However, the engrafted cells mostly differentiated into astrocytes, but not oligodendrocytes in both groups. In the CsA-treated group, a significant decrease in spinal cord lesion volume along with increase in spared myelin and neurons were found compared to the control group. Such histological improvement correlated well with an increase in behavioral recovery. Further study suggested that CsA treatment could inhibit infiltration of T cells and activation of resident microglia and/or macrophages derived from infiltrating monocytes in injured spinal cords, which contributes to the survival of engrafted OPCs and repair of spinal cord injury (SCI).

**Conclusions:**

These results collectively indicate that CsA can promote the survival of engrafted OPCs in injured spinal cords, but has no effect on their differentiation. The engrafted cells mostly differentiated into astrocytes, but not oligodendrocytes. The beneficial effect of CsA on SCI and the survival of engrafted cells may be attributed to its neuroprotective effect.

## Background

Oligodendrocytes are cells that produce myelin in the central nervous system (CNS). They wrap axons of neurons to produce myelin sheaths, provide trophic support and protection for neurons and their axons [1,2]. At least a part of the functional deficit after spinal cord injury (SCI) is attributable to chronic progressive demyelination [3]. Therefore, it seems to be an effective strategy to increase the extent of remyelination by transplanting CNS myelin-forming cells into the injured spinal cord.

Oligodendrocyte precursor cells (OPCs), which are still bipotential in vitro and can differentiate into myelin-forming cells of the CNS under certain conditions. As one of the promising candidate cells, OPCs have been used for treatment of SCI [4-6]. Using a special culture system, we have induced OPCs from rat embryonic spinal cord-derived neural precursor cells (NPCs) [7] and transplanted them into injured spinal cord. However, our study revealed that after being transplanted into the spinal cord, only a small number of the OPCs could survive, and most of them differentiated into astrocytes, but not oligodendrocytes. The mechanisms which caused to this result may be related to a disadvantageous micro-environment in the injured zone created by lipid peroxidation [8-11], an inflammatory reaction [12,13], and/or an immune response against grafts [5,14,15], etc.

Cyclosporin-A (CsA) is an immunosuppressive agent that can depress cellular and humoral immune responses by inhibiting T helper lymphocyte proliferation [9,16]. It can also diminish overproduction of free radicals and lipid peroxidation, which were both observed after acute SCI. CsA does this by inhibiting both the inflammatory reaction and the synthesis of nitric oxide [9,16-20]. Therefore, CsA may act as a neuroprotective agent and be useful in the treatment of acute SCI.

In this study, we transplanted embryonic NPC derived-OPCs which express green fluorescent protein (GFP-OPCs) into injured spinal cords of rats treated with or without CsA, to evaluate whether the drug is beneficial for survival and differentiation of engrafted OPCs and plays a neuroprotective role after SCI.

## Results

### Identification of NPC-induced GFP-OPCs, derived from rat embryonic spinal cord

**Figure 1:**
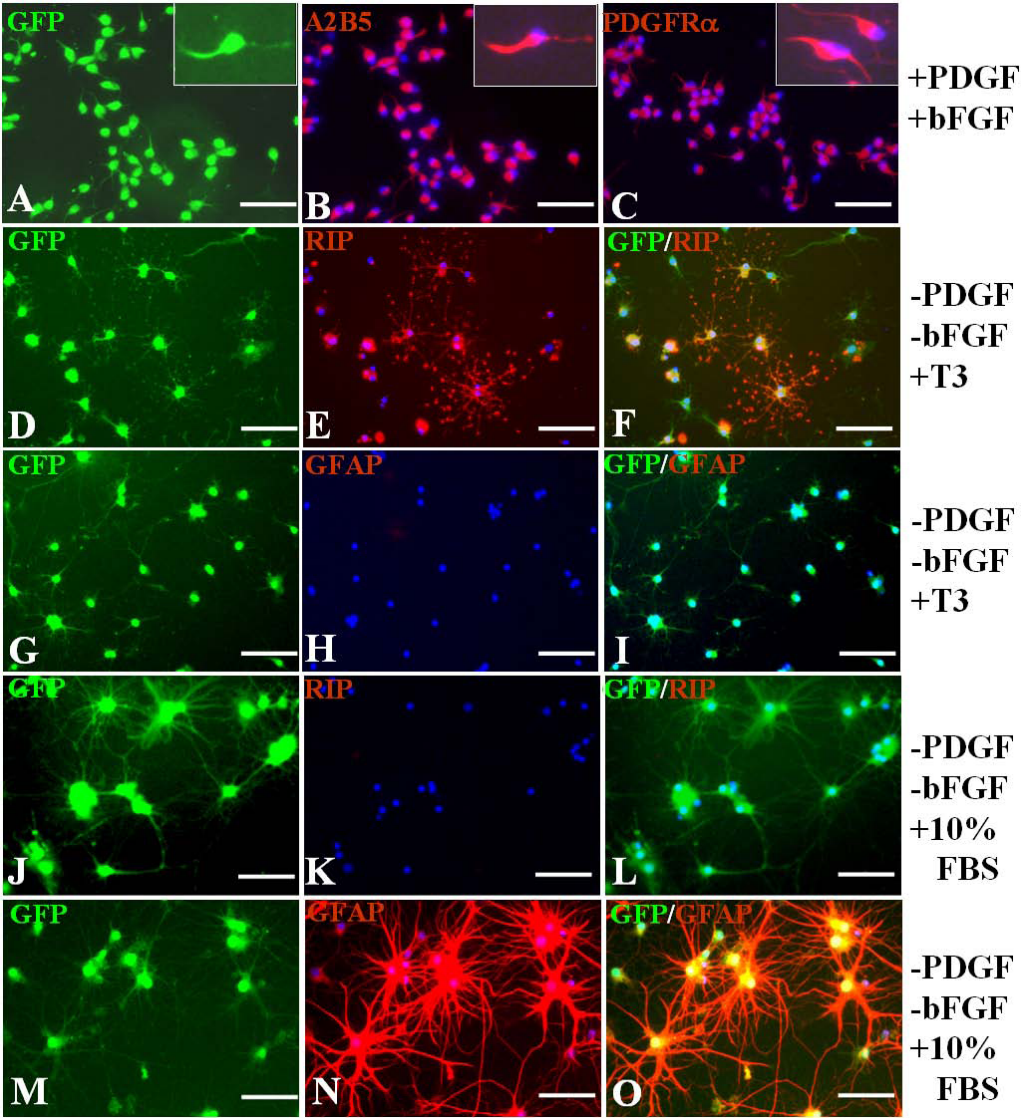
**Identification and differentiation of GFP-OPCs**. The GFP-OPCs induced from spinal cord-derived NPCs were cultured in different media for 5 days. (A~C): In the basal-OPC-medium containing PDGF and bFGF, the cells display bipolar or tri-polar morphology, the typical morphology of OPC (A), more than 95% of cells express both A2B5 (B) and PDGFR (C). Inserts show higher power photographs of OPCs. (D~I) In the medium containing T3 and without PDGF and bFGF, the cells display a multipolar morphology (D, G), more than 95% of the cells express RIP (E, F), and almost no cells express GFAP (H, I).(J~O) In the medium containing 10%FBS without PDGF and bFGF, the cells display the typical process-bearing morphology of astrocytes (J, M), few cells express RIP (K, L) and nearly all cells express GFAP (N, O). Cells in B, C, E, F, H, I, K, L, N and O were counterstained with Hoechst33342 (blue), a nuclear dye. Scale bars: 25 μm.

GFP-OPCs were cultured for 5 days in different media as described in Materials and Methods, to assess their differentiation potential. When oligospheres were triturated into single cells and plated onto coverslips in basal-OPC-medium supplied with PDGF-AA and bFGF (+PDGF, +bFGF), almost all of the cells displayed bipolar or tri-polar morphology, the typical morphology of OPCs (Fig. 1A). Among them, more than 95% of cells expressed both A2B5 and PDGFRα (Fig. 1B, C). In the presence of T3 without PDGF-AA and bFGF (-PDGF, -bFGF, +T3), the cells displayed a multi-polar morphology (Fig. 1D, G). More than 95% of them expressed RIP (Fig. 1E, F), and almost no cells expressed GFAP (Fig. 1H, I). In the presence of 10% FBS (-PDGF, -bFGF, +10%FBS), the cells displayed the typical process-bearing morphology of astrocytes (Fig. 1J, M). Few cells expressed RIP (Fig. 1K, L) and nearly all cells expressed GFAP (Fig. 1N, O).

### Effect of CsA on the survival of engrafted OPCs in injured spinal cord

**Figure 2:**
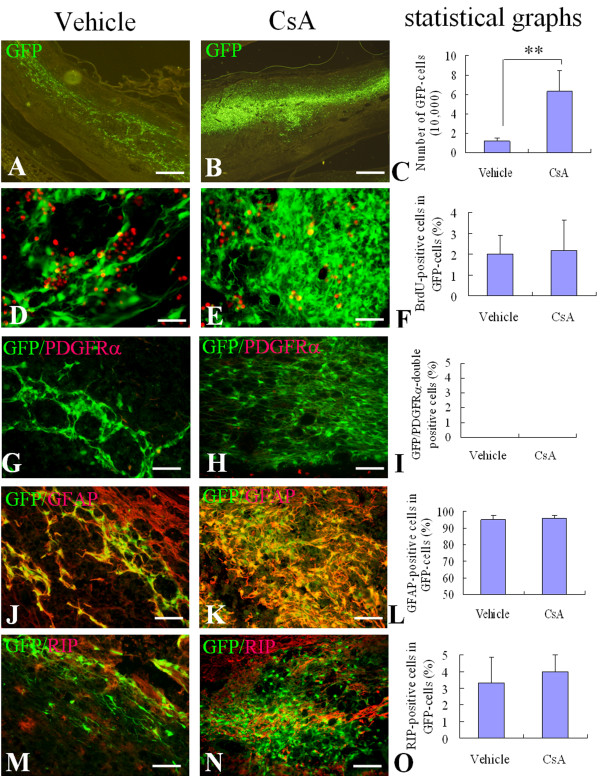
**Survival and differentiation of OPCs after engraftment into the injured adult rat spinal cord for 2 weeks**. Two weeks after cell transplantation, the survival and differentiation of engrafted cells were detected in sagittal sections of injured spinal cord. A, B: Representative pictures show surviving GFP-cells in the vehicle (A) and CsA (B) treated groups. D, E, G, H, J, K: Representative merged images show GFP/BrdU-double positive cells (yellow) in the vehicle (D) and CsA (E) treated groups, GFP/GFAP-double positive cells (yellow) in the vehicle (G) and CsA (H) treated groups, and GFP/RIP-double positive cells (yellow) in the vehicle (J) and CsA (K) treated groups. The statistical graphs show the number of GFP-cells (C), the percentages of BrdU (F), GFAP (I) and RIP (L) positive cells in GFP-cells. Data are given as means ± SD, *n *= 6, ** *p *< 0.01 (*t *test). Scale bar in A, B: 500 μm; in D, E: 20 μm; in G, H, J, and K: 40 μm.

To investigate the survival of engrafted OPCs *in vivo*, we engrafted GFP-OPCs into contusive injured spinal cord at 10 days after SCI. To investigate whether the engrafted GFP-OPCs could survive to the end of the experiment, we detected the engrafted cells by GFP fluorescence at 2 weeks and 6 weeks after transplantation. We also determined the effect of CsA on the survival of the engrafted OPCs. The results showed that the GFP cells could be observed in spinal cords of cell-transplanted rats at 2 weeks and 6 weeks after transplantation. Because it is extremely difficult to discern individual cells at 6 weeks, we chose 2 weeks to count the survived cells and determine their phenotype. Figure 2 shows the representative pictures from the spinal cords after cell transplantation for 2 weeks. Quantitative analysis showed that the average total number of GFP-positive cells present in the spinal cord of vehicle control group and CsA-treated group were 3.02% (12,095 ± 3,364) and 15.77% (63,111 ± 22,149) of the original injected cell number (4 × 10^5^), respectively. There was statistically significant difference between the two groups (*p *< 0.01, Fig. 2A-C). To examine the proliferation of transplanted cells, animals received injections of BrdU before they were sacrificed at 2 weeks after transplantation. We observed few GFP/BrdU-double positive cells in both vehicle and CsA-treated groups. There was no statistically significant difference between the two groups (*p *> 0.05, Fig. 2D-F). These results collectively indicate that the increased numbers of GFP-cells were likely the result of enhanced survival by CsA.

### Effect of CsA on differentiation of engrafted OPCs in injured spinal cord

Next, we investigated whether CsA has any effect on the differentiation of engrafted OPCs. The differentiation of engrafted GFP-OPCs in spinal cords from vehicle- and CsA-treated groups was detected by immunofluorescence staining of PDGFRα, GFAP and RIP, respectively. We found that the most GFP-cells expressed GFAP, only a very small number of GFP-cells expressed RIP and almost no GFP-cells expressed PDGFRα in both groups. Among the total GFP-positive cells, the GFP/GFAP-double positive cells were 95.00% ± 2.59% and 96.00% ± 1.83% in vehicle- and CsA-treated groups, respectively. The GFP/RIP-double positive cells were only 3.33% ± 1.53% and 4.00% ± 1.00% in vehicle- and CsA-treated groups, respectively. The percentage of both markers had no statistically significant difference between the two groups (*p *> 0.05, Fig. 2G-O).

### Effect of OPC transplantation and CsA on infiltration of CD3^+^T cells and activation of resident microglia cells and/or macrophages derived from infiltrating monocytes in the injured spinal cords

**Figure 3:**
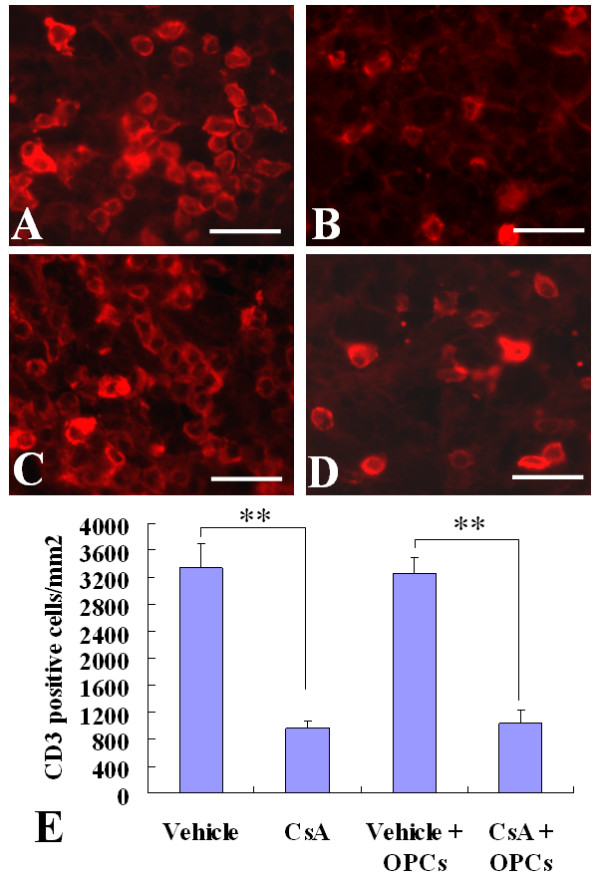
**Infiltration of CD3^+^T cells in the injured spinal cords**. Two weeks after cell transplantation, immunofluorescence labeling showed the CD3^+ ^T cells in injured spinal cords. A~D: Representative pictures show CD3^+ ^cells in the vehicle (A), CsA (B), GFP-OPC transplantation with vehicle (C) or with CsA (D) groups. E: The statistical results. Data are given as means ± SD, *n *= 6, ** *p *< 0.01 (ANOVA). Scale bar 20 μm.

**Figure 4:**
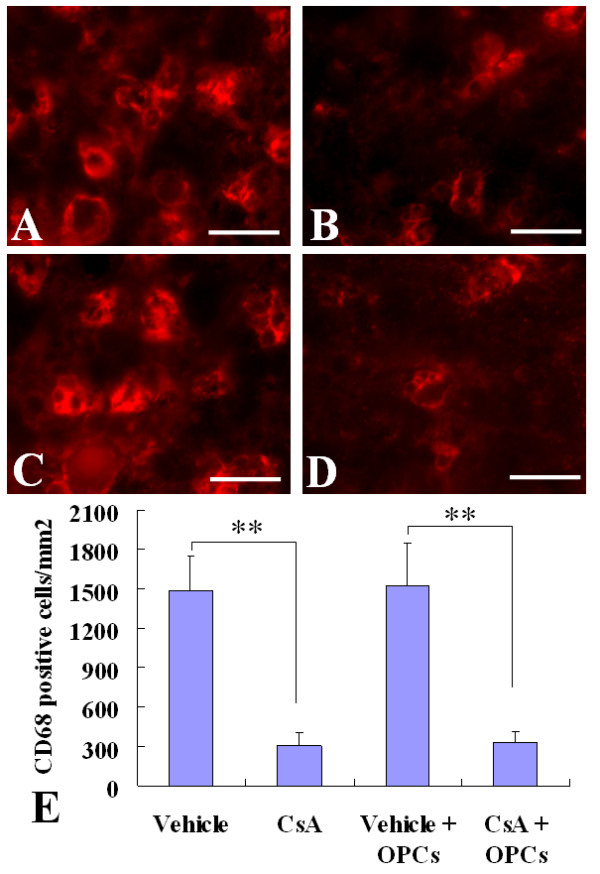
**Activation of microglia and/or macrophages derived from infiltrating monocytes in the injured spinal cords**. Two weeks after cell transplantation, immunofluorescence labeling showed the activated microglia and/or macrophages derived from infiltrating monocytes (CD68^+^) in injured spinal cords. A~D: Representative pictures show CD68^+ ^cells in the vehicle (A), CsA (B), GFP-OPC transplantation with vehicle (C) or with CsA (D) groups. E: The statistical results. Data are given as means ± SD, *n *= 6, ** *p *< 0.01 (ANOVA). Scale bar 20 μm.

To observe the effect of CsA on immune responses, we examined the infiltration of CD3^+^T cells, and activation of resident microglia and/or macrophages derived from infiltrating monocytes in the injured spinal cord at 2 weeks after cell transplantation. The CD3, a specific marker of T lymphocytes, and CD68, a marker of activated resident microglia and macrophages derived from infiltrating monocytes were detected by immunofluorescent staining. The numbers of CD3-positive cells in injured spinal cord of vehicle- and CsA-treated groups were 3,340 ± 355, and 958 ± 116, respectively, and of the OPC transplantation without and with CsA groups were 3261 ± 230, and 1037 ± 191, respectively (Fig. 3). The CD68-positive cells were 1485 ± 262, 309 ± 97, 1519 ± 328, and 326 ± 83, respectively (Fig. 4). The numbers of CD3-positive and CD68-positive cells in CsA-treated groups (with or without OPC transplantation) were significantly lower than those of vehicle-treated groups (*p *< 0.01), while the OPC transplantation had no effect on the infiltration of T cells and activation of microglia and/or macrophages derived from infiltrating monocytes (Fig 3E and Fig 4E).

### Effect of OPC transplantation and CsA on the lesion volume of contused spinal cord

**Figure 5:**
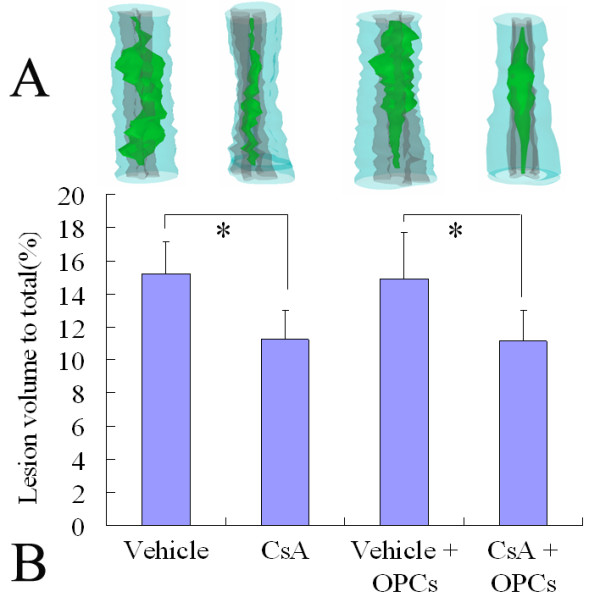
**Three-dimensional reconstruction of lesion volumes at the 7^th ^week after contusive SCI**. A: Representatives of the three-dimensional reconstruction of a 10 mm spinal cord segment from each group containing the lesion cavity (green). The spinal cord contours and white matters are shown in semitransparent blue, and the gray matter is depicted in gray. B: Data are given as means ± SD, *n *= 6, * *p *< 0.05 (ANOVA).

Quantitative analysis of the total lesion volume in whole spinal cords in all groups was performed at the 7^th ^week after SCI. In the vehicle-treated group, the total lesion volume was 15.20% ± 1.97%. In the CsA-treated group, it was 11.23% ± 1.78%. In OPC transplantation with the vehicle-treated group, it was 14.89% ± 2.81%. In OPC transplantation with the CsA-treated group, it was 11.16% ± 1.83%. The total lesion volumes in CsA-treated groups (with or without OPC transplantation) were significantly lower than those of vehicle-treated groups (*p *< 0.05), while there was no difference between the two vehicle-treated groups or the two CsA-treated groups (with or without OPC transplantation) (Fig 5). These results indicate that CsA treatment can significantly decrease spinal cord lesion volume, while OPC transplantation has no effect.

### Effect of OPC transplantation and CsA on the survival of motoneurons in the ventral horn following SCI

**Figure 6:**
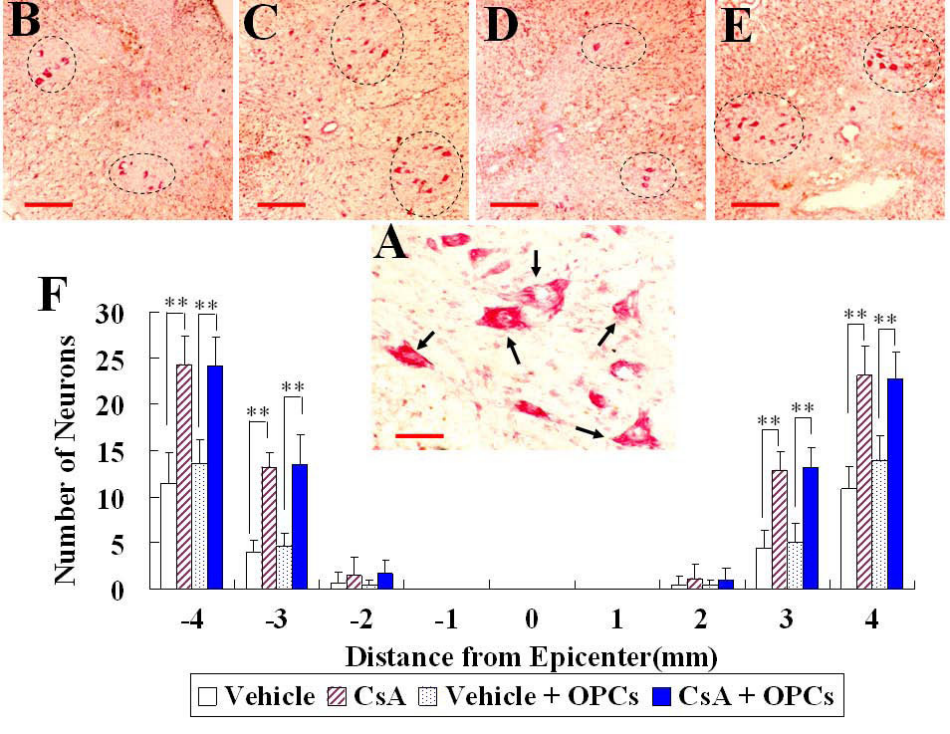
**Survival of motoneurons in the spinal cord ventral horn (VH) at the 7^th ^week after contusive SCI**. A: Neutral Red staining shows VH neurons (arrows) from a CsA treated group in a section 8 mm rostral to the epicenter. B~E: Representative pictures show VH neurons (oval-shaped dotted line) at 3 mm rostral to the injury epicenter in the vehicle (B), CsA (C), GFP-OPC transplantation with vehicle (D) or with CsA (E) groups. F: Comparison of VH neurons among different groups at various distances from the injury epicenter (0) as well as at 1, 2, 3, 4 mm rostral (+) and caudal (-) to it. Data are given as means ± SD, *n *= 6, ** *p *< 0.01 (ANOVA). Scale bar in A: 20 μm, in B and C: 200 μm.

To determine the effect of OPC transplantation and CsA on neuronal survival, the numbers of ventral horn (VH) motor neurons at the injury epicenter as well as at 1, 2, 3, and 4 mm rostral and caudal to the epicenter were counted at the 7^th ^week after injury. As shown in Fig 6, in CsA-treated groups (with or without OPC transplantation), more residual motor neurons were found in the VH at 3 and 4 mm rostral and caudal to the lesion epicenter than in vehicle-treated groups (*p *< 0.01). However, the number of motor neurons showed no statistically significant difference between the OPC transplantation group and the corresponding control (*p *> 0.05).

### Effect of OPC transplantation and CsA on myelin preservation following SCI

**Figure 7:**
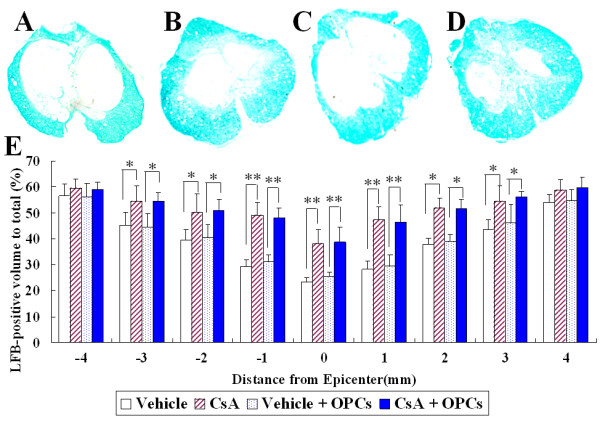
**Quantification of residual myelination in injured spinal cords at the 7^th ^week after contusive SCI**. A~D: Representative pictures show LFB-stained cross-sections of spinal cords taken from the injury epicenter in the vehicle (A), CsA (B), GFP-OPC transplantation with vehicle (C) or with CsA (D) groups. E: Comparison of residual myelination among different groups at various distances from the injury epicenter. Data are given as means ± SD, *n *= 6, * *p *< 0.05, ** *p *< 0.01 (ANOVA).

To investigate the effect of OPC transplantation and CsA on myelin preservation, the extent of residual myelination, stained with Luxol fast blue (LFB), was examined at the injury epicenter at the 7^th ^week after a contusive SCI. Residual myelination was also examined at 1, 2, 3, and 4 mm rostral and caudal to the injury epicenter. As shown in Fig 7, the volume of residual myelin was significantly increased in CsA-treated groups (with or without OPC transplantation), at the epicenter as well as at 1, 2 and 3 mm rostral and caudal to the lesion epicenter compared with that in vehicle-treated groups (with or without OPC transplantation, *p *< 0.01 or 0.05). However, no statistically significant difference was found in the volume of residual myelin between the OPC transplantation group and the corresponding control (*p *> 0.05).

**Figure 8:**
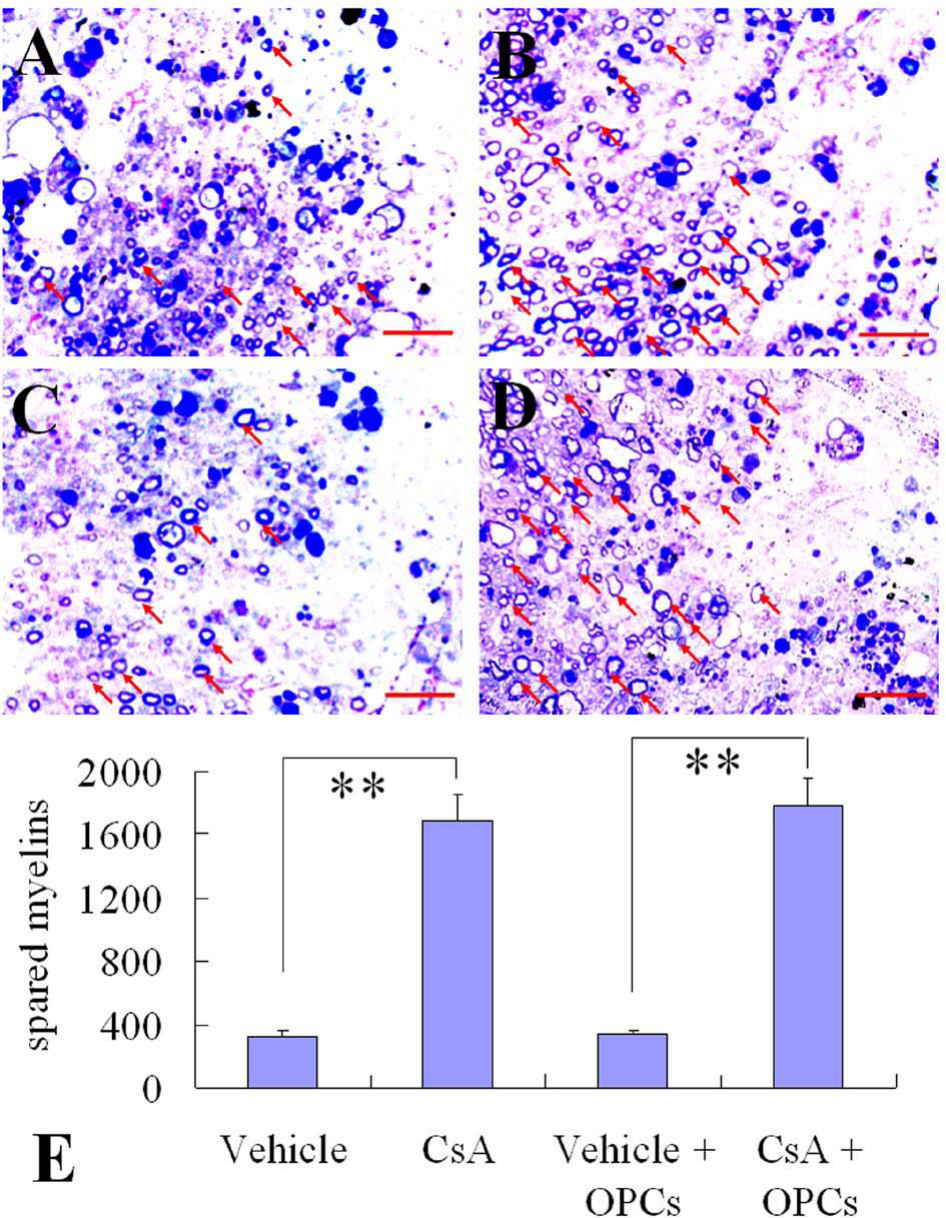
**Quantification of spared myelin in injured spinal cords at the 7th week after contusive SCI**. A-D: Representative photomicrographs of toluidine blue-stained sections taken from the sites in the middle of lesion border and pial border of vehicle (A), CsA (B), GFP-OPC transplantation with vehicle (C) or with CsA (D) groups. Spared myelins (arrows) are more abundant in CsA-treated groups (B and D) compared to the other two groups (A and C). E: Data are given as means ± SD, *n *= 3, ** *p *< 0.01 (ANOVA).

To verify the LFB results, a subset of sections from the injury epicenter of the above groups were stained with toluidine blue. As shown in Fig 8A-D, the spared myelin was more abundant in the CsA-treated groups (with or without OPC transplantation) compared with the vehicle-treated groups (with or without OPC transplantation, Fig 8E, *p *< 0.01).

### Effect of OPC transplantation and CsA on functional recovery in rats after SCI

**Figure 9:**
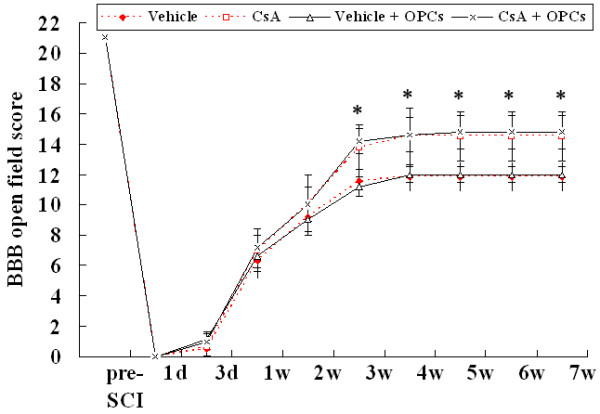
**BBB test showing effects GFP-OPC transplantation and CsA on functional recovery following SCI**. The BBB scores in vehicle, CsA, GFP-OPC transplantation with vehicle (vehicle + OPCs) or with CsA (CsA + OPCs) groups are compared. Data are given as means ± SD, *n *= 12, * *p *< 0.05 (ANOVA).

To observe the effect of OPC transplantation and CsA on functional recovery after SCI, the Basso, Beattie, and Bresnahan (BBB) locomotor rating scale was evaluated at 1 and 3 days, and then weekly up to 7 weeks. As shown in Fig 9, all animals scored 21 points before SCI. At 1 day after SCI, all animals received a score of 0, and at 3 days, a score under 2. In the following days, the locomotor performance substantially improved and reached a plateau at the third week. Starting from the first week, the BBB scores in CsA-treated groups (with or without OPC transplantation) were consistently higher than the other groups. Additionally, the differences between the CsA-treated groups and the other 2 groups were statistically significant starting from the third week and continuing until the 7^th ^week (*p *< 0.05). Notably, no statistically significant difference was found in BBB scores between the OPC transplantation groups and the corresponding controls (*p *> 0.05). These results indicate that the OPC transplantation alone dose not affect the behavior of SCI rats and that CsA-treatment can exert some protective effects leading to functional recovery in SCI rats.

## Discussion

After SCI, many axons remain intact but are rendered useless by loss of their insulating myelin. OPCs are precursors which can differentiate into myelin-forming cells of the CNS. We expected that the OPCs transplanted into injured spinal cord could differentiate into mature oligodendrocytes, induce axonal remyelination and promote functional recovery. However, our results demonstrated that only a small part of the OPCs could survive, and most of them differentiated into astrocytes. Almost no oligodendrocytes could be observed after OPCs were grafted into injured spinal cord. This indicated that the injured spinal cord environment might be unfavorable for the grafted cell survival and differentiation into oligodendrocytes. Therefore, it is of great significance to improve the local microenvironment of injured spinal cord when the OPCs are grafted.

CsA is one of the foremost immunosuppressive agents and has widespread use in tissue and cell transplantation [21-23]. However, it also carries a risk of CNS toxicity due to the drug-induced death of oligodendrocytes and neurons [24]. Therefore, whether the drug is beneficial to survival and differentiation of engrafted NPC-derived OPCs in injured spinal cord remains unknown. This is a question about clinical application because any potential cell transplantation therapy of SCI would require the use of immunosuppressants, and it is therefore important to characterize any effects of these agents might have on transplanted cells. To clarify this question, we used CsA immediately after injury rather than at the time of transplantation. This exposed the cells to an immunosuppressive environment in which immune responses, inflammatory reaction and the synthesis of nitric oxide, which is caused by SCI, could be inhibited [9,16-20].

We observed the survival, proliferation, and differentiation of engrafted OPCs using GFP expressed-OPCs which were induced from GFP-NPCs, derived from GFP transgenic rat embryonic spinal cord. The immunocytochemistry results revealed that the GFP-OPCs used here were highly pure and expressed both A2B5 and PDGFRα, the two commonly used cell surface markers for OPCs [25-27]. They could proliferate, be passaged, differentiate into oligodendrocytes and type-2 astrocytes under certain conditions *in vitro*, and showed classical properties of OPCs [26-28]. Next, the *in vivo *experiments showed that the treatment of CsA resulted in a dramatic increase in the numbers of grafted cells in the injured cord. This suggested that CsA-treatment could enhance the survival of engrafted GFP-OPCs and/or induce their proliferation *in vivo*. To determine whether CsA contributed to the proliferation of transplanted cells, we used the BrdU incorporation assay. We observed few GFP and BrdU-double positive cells in both vehicle and CsA-treated groups. This result indicated that the increased numbers of GFP-expressing cells were likely the result of enhanced survival.

Oligodendrocyte differentiation of engrafted OPCs is vital for OPC transplantation treatment of SCI. Therefore, it is of great significance to clarify whether CsA could affect differentiation of the engrafted OPCs. Thus, the differentiation of engrafted GFP-OPCs in spinal cords was detected by immunofluorescent staining. Unfortunately, we found that the treatment of CsA had no effect on the differentiation of engrafted cells; the exact mechanism is still unclear. Therefore, it is of great significance to clarify the reason behind the phenomenon, and seek new approaches to cause the engrafted OPCs to differentiate into oligodendrocytes rather than astrocytes.

Some previous studies have reported that the OPCs isolated directly from embryonic, neonatal and adult spinal cord, or induced from ES cells could all differentiate into oligodendrocytes after being transplanted into CNS [4-6,29-32]. However, in this study, using embryonic NPC-derived OPCs, we found that few oligodendrocytes could be observed and the majority of engrafted-OPCs differentiated into GFAP-positive astrocytes in both vehicle and CsA-treated groups. However, we have demonstrated that the OPCs derived from NPCs could differentiate into mature oligodendrocytes *in vitro*. We consider that the different source of the OPCs may contribute to the difference between our results with the previous. In addition to cell source, there may be other reasons. For example, in this study, the transplants occurred in animals that had been on CsA for 10 days, which is unlike other studies. However, the detailed mechanism still needs to be further investigated.

To determine whether transplantation of OPCs and/or CsA-treatment could improve recovery of function after SCI, morphological and functional assays were performed. To evaluate the neuroprotective effect of CsA on SCI, we used CsA immediately after injury. Thus, this could exert its neuroprotective effect as early as possible and be suitable for clinical application. We found that in all CsA-treated groups (with or without OPC transplantation), there was a significant decrease in spinal cord lesion volume. Additionally, increases in spared myelin, myelinated axons and neurons were found compared to the vehicle-treated groups. Such histological improvement correlated well with increase in behavioral recovery. However, no histological or behavioral improvement was found in the OPC-transplanted groups compared to the corresponding control groups. These results collectively indicate that the application of CsA may improve myelin preservation. However, it is also possible that more endogenous OPCs were spared and remyelination was improved since the CsA was used immediately after injury.

How does CsA enhance the survival of engrafted OPCs in injured spinal cord and improve spinal cord repair? The previous studies demonstrated that CsA is a selective immunosuppressive agent known for its neuroprotective properties. The neuroprotective role is thought to be attributed to inhibit immune responses, inflammatory reaction and the synthesis of nitric oxide. By these means, CsA may diminish overproduction of self-reactive cells, inflammatory cytokines, free radicals and lipid peroxidation, all of which can be observed after acute SCI [9,16-20]. Because the disadvantageous micro-environment in the injured spinal cord may be triggered by activated microglia and infiltrated lymphocytes [33-36], we supposed that the beneficial effect of CsA on SCI might also be attributed to its effect on immune cells. Notably, we found that CsA could significantly inhibit infiltration of T cells, and activation of microglia and/or macrophages derived from infiltrating monocytes in the injured spinal cords. These results suggest that inhibition of T cell infiltration, microglia and/or macrophage activation may be an important cause, which leads to more survival of engrafted OPCs and improvement of morphological and functional recovery after SCI.

An important observation in the present study is that CsA had a protective effect on SCI. This was consistent with the previous reports [9,16-20], but it seemed to be paradoxical with the results of Rabchevsky and colleagues who used a similar SCI model to test whether CsA could improve functional outcome and tissue sparing. Using careful analysis, they showed absolutely no benefit of CsA on tissue sparing or BBB scores after SCI [37]. However, the CsA administration scheme in their report was very different from ours. In our study, the animals received a daily subcutaneous injection of CsA (10 mg/kg) starting the first day of SCI and continuing until the end of the experiments. In the report of Rabchevsky and co-workers, the rats received a bolus i.p. injection of 20 mg/kg CsA 15 min post-injury and continuous CsA delivery (2.5 mg/day) via an osmotic minipump for only 7 days [37]. Therefore, the differences between their and our results are possible and understandable. However, the exact explanation remains to be determined.

## Conclusions

In conclusion, our study indicates that CsA can promote the survival of engrafted OPCs in injured spinal cords, but has no effect on their differentiation fate. The engrafted cells mostly differentiate into astrocytes, but not oligodendrocytes. The beneficial effect of CsA on SCI and the survival of engrafted cells may be attributed to its neuroprotective effect.

## Methods

### Animals

A total of 76 adult female Sprague-Dawley (SD) rats (200 ~ 250 g) were used in this study. Among them, 4 green fluorescent protein (GFP) transgenic rats (provided by Genome Information Research Center, Osaka University) on gestation day 14.5 were used as the source of NPCs. The other rats were received contusive SCI. All surgical interventions and postoperative animal care were provided in accordance with the Guide for the Care and Use of Laboratory Animals (National Research Council, 1996) and the Guidelines and Policies for Rodent Survival Surgery provided by the Animal Care and Use Committees of Bengbu Medical College.

### Culture of spinal cord-derived GFP-NPCs

Spinal cord-derived GFP-NPCs were prepared according to the methods of Fu et al [38], with appreciable modifications. Briefly, embryonic spinal cords were collected from GFP transgenic E14.5 SD rats. The cells were isolated by mechanical pipetting in Leibovitz's L-15 medium (Gibco, Grand Island, N.Y.). The suspension was filtered through a nylon mesh of 70 μm. After washing, cells were seeded at a density of 1 × 10^5 ^cells/ml, and incubated at 37°C in a humidified 5% CO_2_-95% air atmosphere. The culture medium, referred as basal-NPC-medium, was composed of DMEM/F12 (Gibco), 1% N2 (Gibco), 1% B27 (Gibco), 3 μg/ml heparin (Sigma, St. Louis, MO), and 2 mM glutamine (Gibco), supplemented with 20 ng/ml basic fibroblast growth factor (bFGF, Gibco) and 20 ng/ml epidermal growth factor (EGF, Sigma). At day 3 or 4, one-sixth of the basal-NPC-medium was supplemented. The incubation was extended until day 6, and neurospheres were collected, mechanically dispersed into single cells, and then passaged.

### Induction of GFP-OPCs from GFP-NPCs

The GFP-OPCs induced from GFP-NPCs were prepared according to our previous methods [7,39]. Briefly, freshly dissociated spinal cord-derived GFP-NPCs were seeded at 1 × 10^5^/ml in basal-NPC-medium supplemented with 10 ng/ml bFGF and EGF. The cultures were fed every 2 days by removing one third of the volume of NPC-medium and adding back the same volume of fresh OPC-medium [basal-NSC-medium containing 0.1% bovine serum albumin (BSA) and 10 ng/ml biotin (referred as basal-OPC-medium), supplemented with 10 ng/ml platelet-derived growth factor-AA (PDGF-AA, Chemicon Inc. Temecula, CA) and 10 ng/ml bFGF]. When the majority of cells in the neurospheres migrated out and attached to the bottom of the flask, some necrotic spheres were generated and floated within the medium. To eliminate the necrotic spheres and fragmented cells, the old medium was replaced by fresh OPC-medium. Cells were cultured for another 5~7 days and new spheres were generated, which were referred to as oligospheres. For passaging, the supernatant fluid was gently removed and cell cultures were incubated with 1 ml Accutase solution (Innovative Cell Technologies, Inc., San Diego, CA) for 10 min at 37°C. When the cells started to detach from the bottom of the flask, 3 ml of Hank's buffered salt solution (HBSS) was added to each flask and the cells were detached entirely by gentle agitation. The cell suspension was harvested, gently dispersed using a fire-polished Pasteur pipette and then centrifuged at 134 g for 6 min at room temperature (RT). Dissociated cells were washed in 6 ml HBSS for 8 min at 134 g. Finally, cells were plated at a density of 2 × 10^4 ^cells/cm^2 ^in OPC-medium. On the 7th day after plating, new oligospheres were formed and were again dissociated by Accutase for further passage.

### Differentiation of GFP-OPCs

To induce GFP-OPC differentiation, cells were seeded onto coverslips coated with poly-L-lysine, at a density of 3 × 10^4 ^cells/coverslip. Three groups were set up: OPC-medium only (+PDGF, +bFGF), as the OPC control; OPC-medium without PDGF-AA and bFGF, but with 30 μM tri-iodothyronine (T3, Sigma) (-PDGF, -bFGF, +T3), for oligodendrocytes differentiation; OPC-medium without PDGF-AA and bFGF, but with 10% FBS (-PDGF, -bFGF, +10%FBS), for type 2-astrocytes differentiation. The OPCs in all groups were allowed to differentiate for 5 days.

### Contusive SCI

Contusive SCI was performed using a New York University Impactor, as described previously [5,40]. In brief, rats were anesthetized with pentobarbital (50 mg/kg intraperitoneally) and received a laminectomy at the T9 level. After the spinous processes of T7 and T11 were clamped to stabilize the spine, the exposed dorsal surface of the cord was subjected to a weight drop injury using a 10 g rod (2.5 mm in diameter) dropped at a height of 12.5 mm. Sham-operated rats were laminectormized but not contusion. After the injury, the muscles and skin were closed in layers, and rats were placed in a temperature-and humidity-controlled chamber. Manual bladder emptying was performed three times daily until reflex bladder emptying was established. For CsA treatment, the animals received a daily subcutaneous injection of cyclosporine A (10 mg/kg, Sandimmune; Novartis, East Hanover, NJ) starting the first day of SCI and continuing until the end of the experiments [22]. To prevent infections, all animals were given chloramphenicol daily via the drinking water (50~75 mg/kg).

### Transplantation procedures

After SCI for 10 days, seventy-two rats were divided into four groups, including 12 rats as control (vehicle group), 12 rats only treated with CsA (CsA group), 24 rats for GFP-OPC transplantation without CsA (vehicle + OPCs group) and 24 rats for GFP-OPC transplantation with CsA (CsA + OPCs group). To graft GFP-OPCs 10 days after SCI, rats were anesthetized with pentobarbital (50 mg/kg intraperitoneally), the spinal cord was re-exposed and a small window was opened in the dura. Four injections were made at both left and right laterally from midline, 1 mm cranial and caudal to the lesion site, at a depth of 1.3 mm and 0.6 mm. For cell transplantation, two microliters of GFP-OPCs (1 × 10^5 ^cells) in OPC-medium were injected into each site through a glass micropipette with an outer diameter of 50~70 μm and the tip was sharp beveled to 30~50° at a rate of 0.5 μl/min, as described by Cao et al [5]. And the microinjection system is powered by compressed N2. Thus, a total of 400,000 cells were grafted into each rat. The muscle and skin were sutured and normal saline and antibiotics administered to prevent dehydration and infection. The animals were sacrificed at 2w or 6w post-transplantation.

### Immunocytochemistry

Immunostaining assay for differentiated cells was performed as described previously [7,39]. Antibodies against A2B5 (IgM, 1:100, R&D, Minneapolis, MN), platelet derived growth factor alpha receptor (PDGFRα, 1:100, Neomarkers, Fremont, CA), receptor interaction protein (RIP, 1:25, a gift from Dr. Scott R. Whittemore, University of Louisville) and glial fibrillary acidic protein (GFAP, 1:200, Sigma) were used to identify OPCs, oligodendrocytes and astrocytes, respectively [12,26,27,41].

### Immunohistochemical assay

Two weeks after cell transplantation, the animals were given an overdose of sodium pentobarbital (nembutal; 80 mg/kg, i.p.) and were transcardially exsanguinated with 200 ml physiological saline followed by fixation with 300 ml of ice-cold 4% paraformaldehyde in 0.01 M PBS (pH 7.4). After perfusion, a 2 cm spinal cord segment containing the injury epicenter was removed, postfixed overnight in 4% paraformaldehyde in 0.01 M PBS (pH 7.4), and transferred to 30% sucrose in 0.01 M PBS (pH 7.4) overnight at 4°C. After that the specimen was blocked into segments spaced in 5 mm, from the cervical to the sacral cord. The segments were placed in OCT compound embedding medium (Tissue-Tek, Miles, Elkart, IN, USA) and frozen sections of 20 μm thickness were obtained horizontally or transversely using a cryostat (Leica CM1900, Bannockburn, IL, USA), then thaw-mounted on poly-L-lysine-coated slides (Sigma). For immunohistochemistry, the sections were blocked with 10% normal goat serum (NGS) in 0.01 M PBS (pH 7.4) for 60 min, and monoclonal mouse anti-RIP (1:25), anti-GFAP (1:200), anti-CD3 (1:100; SeroTec, Inc, Raleigh, NC, USA) or anti-CD68(ED1) antibody (1:100; SeroTec, Inc) were applied to the sections overnight at 4°C. On the following day, the sections were incubated for 60 min at 37°C with rhodamine-conjugated goat anti-mouse (1:50; Jackson Immuno Research Lab., West Grove, PA, USA) antibodies, and slides were washed, coverslipped, and examined using an Olympus BX60 microscope.

In both immunocytochemistry and immunohistochemistry assays, primary antiserum omission controls and normal mouse and goat serum controls were used to confirm the specificity of the immunofluorescence labeling

Cell quantification on the spinal cord tissue of transplanted rats was performed in an unbiased stereological manner as described previously [22]. For quantification of cell survival, the total number of GFP-expressing/Hoechst33342-positive cells was counted. Based on our microscopic examination, the size of the cell body (including nucleus) of a grafted GFP-OPC is between 10 and 20 μm. To avoid counting the same cell in more than one section, we counted every fifth section (100 μm apart). We only quantified the GFP cell bodies that contained a nucleus (identified with Hoechst33342). To quantify the differentiation pattern of transplanted cells, we counted the number of GFP-positive cells that were double-labeled with a different cell marker. For quantification of double-positive cells, we chose the spinal cord sections with the highest number of GFP-positive cells. For each cell marker, we stained two tissue sections per rat (at least 100 μm apart). Then we counted the number of GFP and cell marker double-positive cells in 10 random fields per section. On average, 200 - 300 cells were counted per section. For quantification of microglia/macrophages and T cells, the number of positive cells was counted in 10 random fields per section. Two sections from the epicenter spaced ~100 μm apart were analyzed for each animal and then averaged. Data are expressed as cell number per square millimeter. For quantification of aggregated microglia/macrophages, we identified the individual microglia/macrophages by each anatomically demarcated nucleus with the help of computer interfaced digital image analysis system. Only the cell bodies were taken into account.

### Histological analyses

Eight weeks after SCI, the animals were sacrificed as above. Histological analyses were performed as described previously [40]. In brief, the spinal cords were blocked into 10 mm segments, serial 20-μm-thick sections through the entire injury site were cut transversely. Two sets of slides (each set containing serial sections spaced 200 μm apart) were stained with Luxol fast blue (LFB), Neutral Red, respectively, to identify myelinated white matter and residual ventral horn motoneurons. The lesion epicenter was defined as the section containing the least amount of spared white matter. The total and cross sectional area of the spinal cord and the lesion boundary were measured with an Olympus BX60 microscope attached to a Neurolucida system (Microbrightfield Inc., Colchester, VT, USA). An unbiased estimation of the percentage of spared tissue was calculated using the Cavalieri method [42]. The total volume of the lesion area (which included areas of cavitation) was calculated by summing their individual subvolumes [43]. Individual subvolumes of the lesion area were calculated by multiplying the cross-sectional area (A) × D, where D represents the distance between sections (200 μm). The percentage total volume of the injured area was calculated by dividing the total volume of lesion area by the total spinal cord volume [5]. LFB and Neutral Red -stained sections at the lesion epicenter and 1, 2, 3, and 4 mm rostral and caudal to the epicenter were analyzed for myelinated white matter and residual ventral horn motoneurons, respectively. The myelinated white matter was quantified by Image pro-plus 5.1 (Media Cybernetics, Inc, Atlanta, GA, USA) and the number of surviving ventral horn neurons were confirmed by the exhibition of Nissl substance, euchromatic nucleus and nucleolus [44].

### Toluidine blue staining

The toluidine blue staining was performed as described previously [40]. In brief, spinal cord segments were fixed overnight in the solution containing 2% glutaraldehyde and 5% sucrose in 0.1 M sodium cacodylate buffer, pH 7.4, followed by 1% osmium tetroxide in the same buffer for 1 h. The tissue was embedded in Spurr's epoxy resin and cured at 70°C. Transverse semithin sections (1 μm) were stained with a mixture of 1% toluidine blue and 1% sodium borate. For statistical analysis, the numbers of spared myelins were calculated in four random 10 × 40-fold microscope views (about 67,500 μm^2 ^per view) in the middle of lesion border and pial border.in the dorsal, lateral, and ventral columns.

### BBB locomotor rating scale

Behavioral assessments were performed using the Basso, Beattie, and Bresnahan (BBB) locomotor rating scale, a 21-point scale (0~21) based on the observation of hind-limb movements of a rat freely moving in an open field [45,46]. The BBB was evaluated at 1, 3 days, 1, 2, 3, 4, 5, 6 and 7 weeks after injury. During the evaluation, animals were allowed to walk freely on the open-field surface for 4 min while being observed by two scorers lacking knowledge of the experimental groups.

### In vivo 5-Bromo-2-deoxyuridine (BrdU) incorporation labelling and detection

Rats received 9 injections of BrdU (50 mg/kg, i.p.; three injections per day for 3 days) and were sacrificed 2 hours after the last injection. The animals were perfused transcardially, and tissues were collected as described above. Then, the slides were processed for immunostaining against the BrdU antibody. The sections were washed with PBS, incubated in 2N HCl and 1% Triton X-100 for 15 min at room temperature, and washed with 0.1 M sodium borate in PBS for 10 min. The slides were blocked with 1% BSA, 5% non-fat milk, and 0.3% Triton X-100 in PBS for 1 hour at room temperature and incubated with a mouse anti-BrdU antibody (1:100; BD Bioscience, Franklin Lakes, NJ) in the same blocking solution for overnight at 4°C. The slides were treated with rhodamine-conjugated goat anti-mouse antibody as described above. The images were taken using an Olympus BX60 microscope.

### Statistical analyses

For BBB locomotor rating scale, the data were analyzed by two-way repeated measures ANOVA followed by post-hoc analyses. For the other data, the paired data were analyzed by two-tailed Student's *t*-test, the data with three or more samples were analyzed by one way ANOVA followed by Student-Newman-Keuls tests of multiple comparisons to determine whether there were significant differences between individual groups. All differences were considered significant at *p *< 0.05.

## Authors' contributions

JGH designed the experiments and wrote the manuscript. HZL and YXW carried out the cell culture, contusive spinal cord injury model, cell transplantation and immunoassays. JSZ and FCW carried out histological analyses, behavioral assessments and the statistical analysis. All authors read and approved the final manuscript.
